# The Effect of Impartial Beneficence on Bystander Cooperation Behavior: The Roles of Social Perception and Impartial Beneficence Personality

**DOI:** 10.3390/bs15060718

**Published:** 2025-05-23

**Authors:** Xiaodan Xu, Yingjun Zhang, Ming Yu, Hongju Li, Feng Zhang, Yan Xu

**Affiliations:** 1 School of Arts and Communication, Beijing Normal University, Beijing 100875, China; 2 Mental Health Education and Counseling Center, Beijing Normal University, Beijing 100875, China; 3 Ideological and Political Work Team Training and Research Center, Beijing Normal University, Beijing 100875, China; 4 Psychological Research and Counseling Center, Southwest Jiaotong University, Chengdu 610031, China; zhangfpsy@swjtu.edu.cn; 5 Beijing Key Laboratory of Applied Experimental Psychology, National Demonstration Center for Experimental Psychology Education, Faculty of Psychology, Beijing Normal University, Beijing 100875, China

**Keywords:** impartial beneficence, cooperative behavior, warmth, competence, impartial beneficence personality

## Abstract

Previous research on utilitarianism has focused predominantly on instrumental harm while neglecting the investigation of impartial beneficence. This study designed three progressive experiments (total *n* = 1378) to explore how impartial beneficence influences bystander cooperation behavior and to uncover the underlying mechanisms and boundary conditions. The findings indicate that (1) compared with a partial agent, an impartial agent reduces bystander cooperation behavior; (2) impartial beneficence affects bystander cooperation behavior by influencing bystanders’ perception of warmth rather than competence; and (3) a bystander’s impartial beneficence personality plays a moderating role in the mediation model, and this moderating effect occurs in the pathways from impartial beneficence to bystander cooperation behavior, perception of warmth, and perception of competence.

## 1. Introduction

Utilitarianism aims to maximize overall benefits (Bentham, 1983; de Lazari-Radek & Singer, 2017). A 2D model of utilitarianism highlights two fundamentally independent dimensions of utilitarianism, namely, instrumental harm (IH) and impartial beneficence (IB). Previous research on utilitarian moral judgments has focused predominantly on instrumental harm (Rom et al., 2017; Everett et al., 2016), while neglecting the attention given to impartial beneficence. From the perspective of impartial beneficence, if the greatest happiness is to be achieved, one must be prepared to make sacrifices even for one’s greatest enemies (Kahane et al., 2018). Impartial beneficence implies an individual’s willingness to sacrifice the interests of peers for the greater interests of the larger group, which may be detrimental to cooperative behavior. In view of this, the present study aims to explore how impartial beneficence influences bystander cooperation behavior through three progressive experiments, gradually uncovering the underlying mechanisms and boundary conditions.

When we enter into close interpersonal partnerships with partners and family members, we anticipate undertaking certain special obligations and expect the same obligations from them. People desire to assist their loved ones when they are in need and, similarly, hope for support from their loved ones when they themselves are in need. The Relational Regulation Theory of Morality (Rai & Fiske, 2011) suggests that behavior that does not conform to relational norms constitutes a moral transgression. However, impartial beneficence utilitarianism conflicts with relational obligations. According to the definition of impartial beneficence, utilitarianism advocates sacrificing the interests of close individuals for the benefit of more distant individuals (Kahane et al., 2015, 2018). Previous research, however, has concluded that people do not support decisions on the basis of impartial beneficence (Berman et al., 2018). Furthermore, morality as cooperation theory views relational obligations as a universal morality (Curry et al., 2019) and morality as a solution to recurring cooperation problems in human social life (Curry, 2016). Individuals who are deemed immoral are also considered unsuitable for cooperation. Thus, it is hypothesized that individuals who violate relational obligations will decrease bystander cooperative behavior. In other words, when bystanders observe an individual acting impartially and without bias in a task, the bystanders may perceive the individual to be less moral and, thus, the bystanders would be unwilling to cooperate with the individual. Specifically, partial agent who adhere to impartial beneficence nonutilitarian moral judges tend to exhibit relational obligations with respect to providing assistance (Marshall et al., 2020) and protective behaviors (Lee & Holyoak, 2020), with the pursuit of interpersonal relationships and in-group intuitions serving as necessary motives for their morality (Scheffler, 2010). In contrast, impartial agents who make impartial beneficence and utilitarian judgments often emphasize assessment and calculation over norms and intuitions. Although impartial beneficence utilitarian philosophy embodies fairness and respect for interests, utilitarian judges still argue that one should engage in actions that yield the best outcomes (Everett et al., 2018), which may be perceived by bystanders as immoral and unsuitable for cooperation. Therefore, the following hypothesis is proposed:

**Hypothesis** **1.** *Compared with partial beneficence nonutilitarian moral judgments, impartial beneficence utilitarian moral judgments decrease bystander cooperation behavior.*

Impartial beneficence utilitarianism violates people’s moral intuitions regarding relational obligations. At the core of moral psychology lies the use of normative models of social relationships to guide the assessment of one’s own and others’ motivations behind judgments and behaviors, which include speech, emotions, attitudes, and intentions (Rai & Fiske, 2011). Behaviors that do not conform to relational norms are considered moral transgressions that may lead to impartial agents being perceived as less warm. According to the innocent sinner hypothesis (Stellar & Willer, 2018), immoral information reduces bystanders’ perception of the competence of impartial agents.

On the basis of the partner selection model of moral reciprocity (Baumard et al., 2013; Barclay, 2016), individuals select potential partners and exhibit high levels of cooperation on the basis of trait-level information, such as their moral character (Shinohara et al., 2019) and adherence to moral norms (Tang et al., 2022). According to the Behaviors from Intergroup Affect and Stereotype Map (BIAS Map; Cuddy et al., 2007; Guan, 2009) discussed earlier, warmth is the primary dimension of perception, whereas competence is a secondary dimension. Higher perceptions of warmth promote proactive facilitating behaviors toward others, whereby beneficence goals, such as cooperation, support, assistance, and protection, reflect a positive emotional orientation, while higher perceptions of competence promote reactive facilitating behaviors, such that passive behavioral goals, adoption of cooperation, or facilitation of long-term benefits or shared goals reflect an instrumental orientation. In other words, compared with impartial agents, partial agents are perceived as warmer and more competent, which promotes greater cooperative behavior from bystanders. Therefore, this study proposes the following research hypotheses:

**Hypothesis** **2a.** *Perception of warmth mediates the influence of impartial beneficence judgment on bystanders’ cooperative behavior.*

**Hypothesis** **2b.** *Perception of competence mediates the influence of impartial beneficence judgment on bystanders’ cooperative behavior.*

On the basis of the behavior from the BIAS map (Cuddy et al., 2007; Guan, 2009), it is well established that warmth is the primary dimension of social perception, whereas competence is a secondary dimension. From evolutionary perspectives such as the intentionality/competence interpretation of the warmth-first effect (Wojciszke & Abele, 2008), the dual-benefit perspective (Abele & Wojciszke, 2007), and the differential distribution of warmth and competence (Eisenbruch & Krasnow, 2022), it is evident that evaluations of warmth play a more crucial role in behavioral responses than do evaluations of competence. Therefore, this paper proposes Hypothesis 2c.

**Hypothesis** **2c.** *The mediating role of perception of warmth on the effect of impartial beneficence judgment on bystanders’ cooperative behavior is greater than that of perception of competence.*

The Interplay of Personality and Social Relationships (PERSOC) model serves as a unified framework for understanding the interaction between personality and social relationships (Back et al., 2011). According to the PERSOC framework, impartial beneficence personality encompass both individual and relational dispositions. In dyadic relationships, impartial beneficence personality influences individuals’ interpersonal perception of moral judgment in impartially beneficence utilitarian dilemmas and shape their corresponding behaviors. Previous research has identified personality traits as antecedent variables related to utilitarian judgments and decisions. For example, antisocial personality, psychoticism, and Machiavellianism are positively correlated with utilitarian judgment (Bartels & Pizarro, 2011), agreeableness is positively related to non-utilitarian judgment (Smillie et al., 2021). Additionally, studies have indicated that individuals’ utilitarian personality are significant factors in utilitarian judgment (Everett et al., 2018; Navajas et al., 2021; Park et al., 2023). Individuals’ impartial beneficence personality are positively correlated with their perception of the correctness of impartial beneficence utilitarian behaviors relative to nonutilitarian behavior, i.e., the greater an individual’s impartial beneficence personality, the more they perceive impartial beneficence behavior as appropriate compared with nonutilitarian behavior (Everett et al., 2018). On the basis of the above, this paper proposes Hypothesis 3.

**Hypothesis** **3.** *The bystander’s impartial beneficence personality plays a moderating role in the mediation model, and this moderating effect occurs on the pathways from impartial beneficence judgment to bystanders’ cooperative behavior, as well as to perceptions of warmth and competence.*

This research employed three progressive experiments to explore how impartial beneficence influences bystander cooperation behavior. The relationship between these experiments follows a logical, building sequence.

Experiment 1 focused on establishing the fundamental relationship between impartial beneficence and bystander cooperation behavior. We tested whether exposure to impartial versus partial moral agents affects bystanders’ willingness to cooperate. Experiment 2 built upon Experiment 1 by investigating the underlying mechanisms. This experiment delved into the internal processes, explaining “why” the effect observed in Experiment 1 occurs. Experiment 3 extended the previous experiments by introducing boundary conditions, as follows: bystanders’ own impartial beneficence personality traits moderate the mediation model. This experiment determined “when” and “for whom” this effect would be strengthened or weakened. Collectively, these three experiments form a comprehensive research progression, as follows: from phenomenon confirmation (Experiment 1) and mechanism exploration (Experiment 2) to boundary condition investigation (Experiment 3), systematically revealing the complete picture of how impartial beneficence influences bystander cooperation behavior.

## 2. Experiment 1

### 2.1. Method

#### 2.1.1. Participants

Using G*Power 3.1 software, with the statistical power (1 − *β*) set at 0.8, a significance level of 0.05, and a medium effect size, where *f* = 0.25, it was calculated that 128 participants were needed. On the Credamo platform, 530 participants (136 males and 394 females; *M*_age_ = 31.58, *SD*_age_ = 9.26) were recruited and randomly assigned to either the impartial agent group (*n* = 265) or the partial agent group (*n* = 265). Upon completion, the participants received a fee of CNY 1.

#### 2.1.2. Experimental Design

Adopting a between-subjects experimental design with a single factor (i.e., impartial agent vs. partial agent), the dependent variable is the number of investment tokens in a public goods task.

#### 2.1.3. Experimental Materials and Tools

Manipulation of Impartial Beneficence (Hughes, 2017): Participants were randomly assigned to either a partial agent or impartial agent condition. All participants read a scenario about a firefighter (Zhang Li) who must choose between saving his mother or a peace negotiator whose work could save many future lives. Partial Agent Condition: participants were informed that their partner (Person A) believes Zhang Li should save his mother, with the justification that filial responsibility outweighs potential future lives saved. Impartial Agent Condition: participants were informed that their partner (Person A) believes Zhang Li should save the peace negotiator, with the justification that maximizing lives saved outweighs familial obligations, see Table 1.

Measurement of Bystander Cooperation Behavior: public goods task (Bostyn & Roets, 2017). In the next phase, you will participate anonymously with three other partners in a following decision-making game.

Each member of the group is allocated 100 game tokens. The group members work together to complete a common project. Each member decides how many tokens they will invest in this project, with investment amounts ranging from 1 to 100 tokens (inclusive). The project’s earnings are calculated as follows: the total number of tokens invested by all four group members is summed and then multiplied by 2 to determine the overall project earnings. These total earnings are then evenly distributed among the four group members. Your final total token earnings are equal to your average token earnings plus the tokens you did not invest.

Baseline: How many tokens do you decide to invest in the decision-making game?

How many tokens do you decide to invest in the decision-making game when playing with Partner A?

As an indicator of cooperative behavior, a greater number of tokens invested by the participant signifies a higher level of cooperation.

Demographic variables: Age, gender, educational background, and subjective socioeconomic status.

#### 2.1.4. Experimental Procedure

First, the participants completed the informed consent process. Second, the participants completed the baseline measurement for the public goods task. The participants were then randomly assigned to one of two experimental conditions and completed the public goods task. Finally, the participants completed the demographic questionnaire.

### 2.2. Results

**Figure 1:**
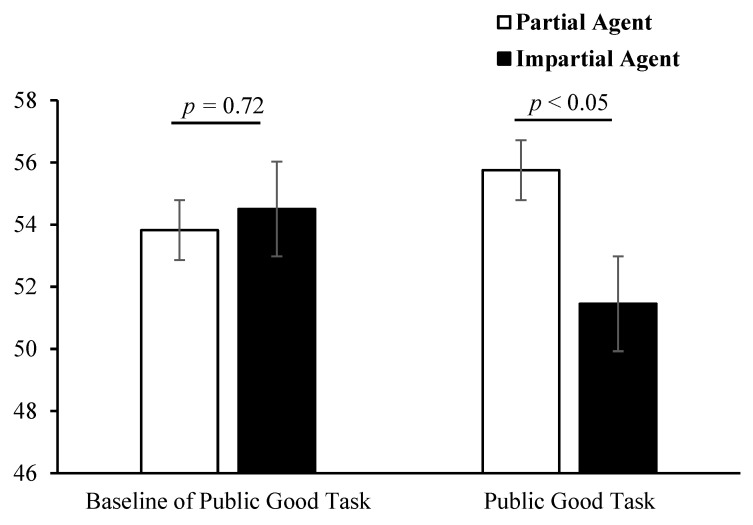
The effects of impartial beneficence on the baseline of the bystanders’ public goods task and the number of tokens invested in the public goods task in Study 1.

Using independent-samples *t* tests, separate analyses were conducted on the number of tokens invested in the public goods task by bystanders and the baseline measurements of the public goods task. The results revealed that the number of tokens invested in the public goods task by bystanders toward those in the impartial agent group was significantly lower than that in the partial agent group (*M*_impartial_ = 51.45, *SD*_impartial_ = 25.92; *M*_partial_ = 55.75, *SD*_partial_ = 22.65, *t*(528) = −2.04, *p* < 0.05). However, no significant difference was found in the baseline measurements of the public goods task between these two groups (*M*_impartial_ = 54.50, *SD*_impartial_ = 22.34; *M*_partial_ = 53.82, *SD*_partial_ = 21.63, *t*(528) = 0.35, *p* = 0.72). See Figure 1.

## 3. Experiment 2

### 3.1. Method

#### 3.1.1. Participants

This study recruited 399 participants (131 males, 268 females; *M*_age_ = 30.14, *SD*_age_ = 8.85, with 1 person not reporting their age) through the sample service of the Credamo (https://www.credamo.com/#/, accessed on 28 April 2023) platform. They were randomly assigned to either the impartial agent group (*n* = 200) or the partial agent group (*n* = 199). The participants were compensated with a fee of CNY 1.

#### 3.1.2. Experimental Materials and Tools

Manipulation of Impartial Beneficence: (Hughes, 2017): see Table 2.

Measurement of Bystander Cooperation Behavior. The trust task (Bostyn & Roets, 2017) was employed. You need to participate in the following single-decision game anonymously with another partner, A (code name). In this game, you are the investor and have 10 game tokens available for investment. Your investment target is A. Your investment amount can range from 0 to 10 and must be an integer (0 represents no investment). Once you decide to invest a portion of your game tokens, A will receive an amount equal to three times your investment. A then has the option to return any number of tokens to you.

Social Perception: With reference to the study by Everett et al. (2018) on bystanders’ perceptions of utilitarian moral judges, how warm do you perceive A to be? Rate on a 7-point scale, where 1 represents not very warm and 7 represents very warm. Additionally, how competent do you perceive A to be? Rate on a 7-point scale, where 1 represents very incompetent and 7 represents very competent.

Demographic Variables: Same as in Experiment 1.

#### 3.1.3. Experimental Procedure

After completing the informed consent and baseline measurement of the trust task on the Credamo platform, the participants were randomly assigned to either the impartial agent grout or partial agent group. The participants subsequently completed assessments of warmth and competence, a single-trial trust task, and provided demographic information.

### 3.2. Results

First, a correlation analysis was conducted among the variables. Significant negative correlations were found between moral judgments and both warmth and the number of investment tokens in the trust task, with correlation coefficients ranging from −0.51 to −0.30 and *ps* < 0.001. The results are presented in Table 3.

Independent sample *t* tests were conducted to analyze warmth and competence separately. The results revealed that there was a significant difference in perceived warmth between the impartial agent group and partial agent group (*M*_impartial_ = 3.76, *SD*_impartial_ = 1.74; *M*_partial_ = 5.50, *SD*_partial_ = 1.14, *t*(397) = −11.85, *p* < 0.001), whereas the difference in perceived competence was not significant (*M*_impartial_ = 4.77, *SD*_impartial_ = 1.34; *M*_partial_ = 4.59, *SD*_partial_ = 1.12, *t*(397) = 1.49, *p* = 0.14), indicating that impartial beneficence judgments significantly affect perceived warmth but not perceived competence.

Using independent-sample *t* test to analyze the number of investment tokens and the baseline performance in the trust task among bystanders, the results revealed significant differences in the number of investment tokens of bystander in the trust task toward the impartial beneficence agent and the partial agent (*M*_impartial_ = 4.39, *SD*_impartial_ = 2.97; *M*_partial_ = 6.14, *SD*_partial_ = 2.71, *t*(397) = −6.17, *p* < 0.001). However, there was no significant difference in the baseline performance of the trust task (*M*_impartial_ = 5.81, *SD*_impartial_ = 2.69; *M*_partial_ = 5.67, *SD*_partial_ = 2.93, *t*(397) = 0.53, *p* = 0.60). This finding suggests that the number of investment tokens in the trust task among bystanders is influenced by impartial beneficence.

On the basis of the correlation analysis, we further examined the mediating role of perceived warmth in the influence of impartial beneficence (1 = impartial agent, 0 = partial agent) on bystanders’ cooperative behavior. Controlling for gender, age, subjective social class, and the baseline performance of the trust task, we conducted a mediation analysis using the Process plugin (Model 4) in IBM SPSS 25, with a bootstrap setting of 5000 iterations. A significant mediation effect was indicated if the 95% confidence interval (CI) did not contain zero (Preacher & Hayes, 2004, 2008).

**Figure 2:**
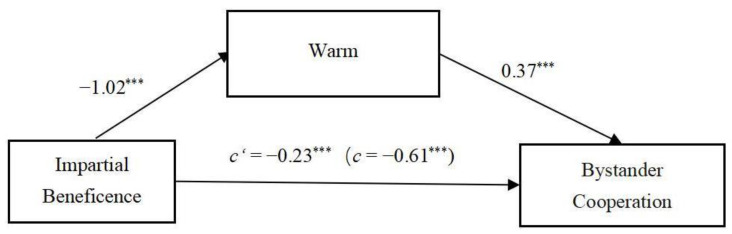
The mediation model in Study 2. 1 = impartial agent, 0 = partial agent. *** *p* < 0.001.

The results indicated that the direct effect of impartial beneficence on bystanders’ cooperative behavior was significant, with a 95% CI = [−0.41, −0.05]. Additionally, the mediating effect of perceived warmth on the relationship between impartial beneficence and bystanders’ cooperative behavior was also significant, with a 95% CI = [−0.49, −0.27]. See Figure 2.

## 4. Experiment 3

### 4.1. Method

#### 4.1.1. Participants

In this study, 449 participants (155 males and 294 females; *M*_age_ = 29.75, *SD*_age_ = 8.12) were recruited through the sample service of the Credamo (https://www.credamo.com/#/, accessed on 1 May 2023) platform. They were randomly assigned to either the impartial agent group (*n* = 225) or the partial agent group (*n* = 224). Upon completion, the participants received a fee of CNY 2.

#### 4.1.2. Experimental Materials and Tools

Manipulation of Impartial Beneficence: Same as in Experiment 2.

Measurement of Bystander Cooperation Behavior. Same as in Experiment 2.

Social Perception: Same as in Experiment 2.

Demographic Variables: Same as in Experiment 1.

Impartial Beneficence Personality: The impartial beneficence dimension of the Oxford Utilitarianism Scale (Kahane et al., 2018) consists of five items, each scored on a 7-point Likert scale (1 = strongly disagree to 7 = strongly agree). The participants were asked, “In the following hypothetical moral scenarios, to what extent do you agree with these statements? Please select the number that best describes your position.” In this study, the consistency coefficient for this scale was 0.76.

#### 4.1.3. Experimental Procedure

After providing informed consent on the Credamo platform, completing the baseline trust task, and measuring impartial beneficence personality, the participants were randomly assigned to either the impartial agent group or partial agent group. The participants subsequently completed assessments regarding warmth, competence, a single trust task, and demographic information.

### 4.2. Results

First, correlation analyses were conducted among the independent variable, dependent variable, mediating variable, moderating variable, and control variables. Impartial beneficence was significantly negatively correlated with warmth, competence, and the number of investment tokens in the trust task, with *r* values ranging from −0.51 to −0.28 and a *ps* < 0.001. The results are presented in Table 4.

After conducting an independent samples *t* test to analyze impartial beneficence personality, it was found that there was no significant difference in the impartial beneficence personality between the impartial agent group and the partial agent group (*M*_impartial_ = 3.53, *SD*_impartial_ = 1.30; *M*_partial_ = 3.41, *SD*_partial_ = 1.23, *t*(447) = 1.05, *p* = 0.29). There was a significant difference in perceived warmth between bystanders toward the impartial agent group and the partial agent group (*M*_impartial_ = 3.22, *SD*_impartial_ = 1.05; *M*_partial_ = 3.88, *SD*_partial_ = 0.75, *t*(447) = −0.75, *p* < 0.001), whereas there was no significant difference in perceived competence (*M*_impartial_ = 3.49, *SD*_impartial_ = 0.94; *M*_partial_ = 3.51, *SD*_partial_ = 0.87, *t*(447) = −0.27, *p* = 0.79). This finding indicates that impartial beneficence significantly affect perceived warmth but not perceived competence. There was a significant difference in the number of investment tokens in the trust task between bystanders toward the impartial agent group and the partial agent group (*M*_impartial_ = 5.27, *SD*_impartial_ = 3.0; *M*_partial_ = 6.57, *SD*_partial_ = 2.52, *t*(447) = −4.99, *p* < 0.001), whereas there was no significant difference in the baseline trust task (*M*_impartial_ = 6.43, *SD*_impartial_ = 2.54; *M*_partial_ = 6.19, *SD*_partial_ = 2.62, *t*(447) = 0.96, *p* = 0.34). This suggests that the number of investment tokens in the trust task by bystanders is influenced by impartial beneficence.

Using impartial beneficence as the independent variable X, bystanders’ investment tokens in the trust task as the dependent variable Y, and warmth and competence as the mediating variables M, a mediation effect analysis was conducted using the bootstrap method through the PROCESS (Model 4) plugin in IBM SPSS 25 while controlling for gender, age, subjective social class, and the baseline of the trust task. The bootstrap was set to 5000 iterations, and a 95% confidence interval (CI) estimate not containing zero indicates a significant mediation effect (Preacher & Hayes, 2004, 2008). The results indicated that there was not significant direct effect from impartial beneficence to bystanders’ cooperative behavior, with a 95% CI = [−0.27, 0.03], bu a significant indirect effect, with a 95% CI = [−0.52, −0.24]. The mediating effect of perceived warmth between impartial beneficence and bystanders’ cooperative behavior was significant, with a 95% CI = [−0.49, −0.25], whereas the mediating effect of competence between impartial beneficence and bystanders’ cooperative behavior was not significant, with a 95% CI = [−0.06, 0.03].

**Figure 3:**
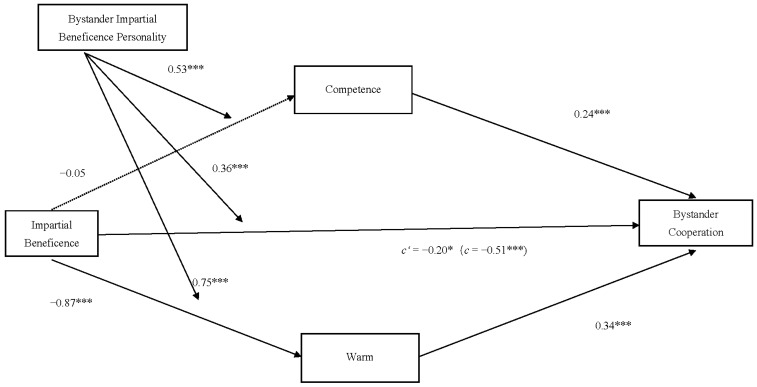
The moderated mediation model in Study 3. 1 = impartial agent and 0 = partial agent. Note: * *p* < 0.05, *** *p* < 0.001. Specifically, bystander’s impartial beneficence personality has a significant moderating effect on the influence of impartial beneficence on bystanders’ cooperative behavior, 95% CI = [0.22, 0.51].

A moderated mediation effect test was conducted using PROCESS (Model 8) (Hayes, 2013). With controls for gender, age, subjective social class, and the baseline of the trust task, impartial beneficence was treated as the independent variable X (with impartial agent coded as 1 and partial agent coded as 0); bystanders’ investment tokens in the trust task as the dependent variable Y; warmth and competence as the mediating variables M; and bystander’s impartial beneficence personality as the moderating variable W. The bootstrap analysis results indicate that the moderated mediation effect was established in the influence of impartial beneficence on bystanders’ cooperative behavior, as illustrated in Figure 3.

**Figure 4:**
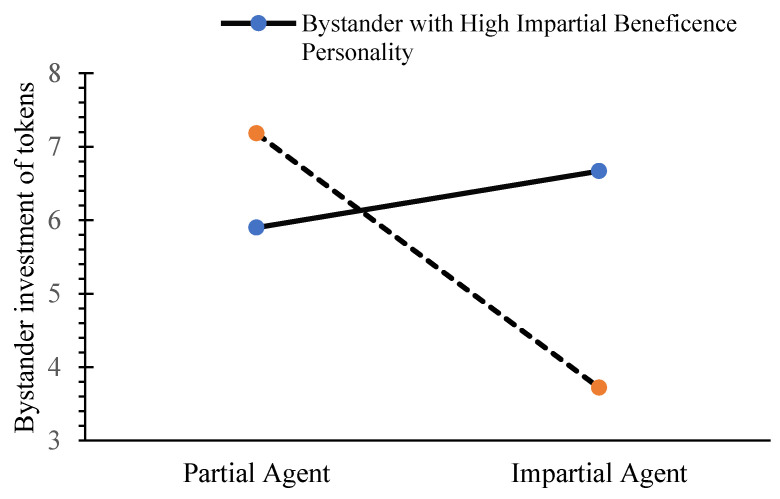
Moderating effect of bystanders’ impersonal beneficence personality on the relationship between impartial beneficence and bystander cooperation behavior.

When bystanders have a higher impartial beneficence personality, impartial beneficence do not significantly predict bystanders’ cooperative behavior, 95% CI = [−0.03, 0.35]. When bystanders have a lower impartial beneficence personality, impartial beneficence negatively predict bystanders’ cooperative behavior, 95% CI = [−0.79, −0.33]. This is illustrated in Figure 4.

**Figure 5:**
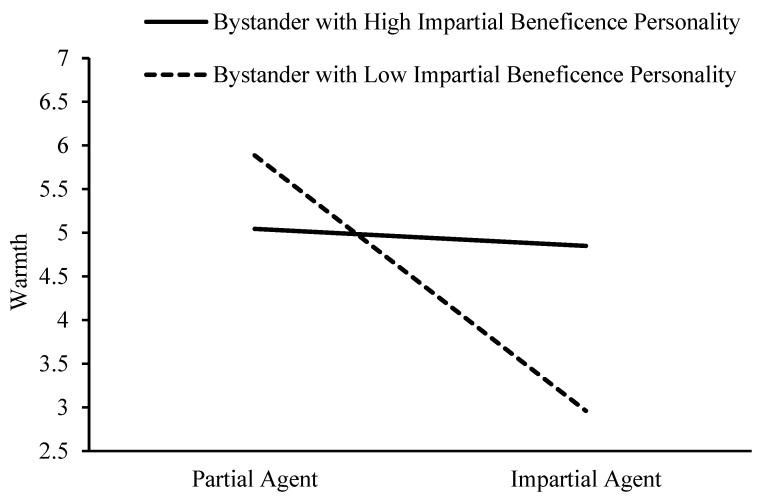
The moderating effect of bystanders’ impartial beneficence personality on the relationship between impartial beneficence and perception of warmth.

The impartial beneficence personality of bystander has a significant moderating effect on the influence of impartial beneficence on perception of warmth, 95% CI = [0.60, 0.90]. When bystanders exhibit higher impartial beneficence personality, impartial beneficence do not significantly predict perception of warmth, 95% CI = [−0.33, 0.09]. When bystanders display a lower impartial beneficence personality, impartial beneficence impartial negatively predict perception of warmth, 95% CI = [−1.83, −1.40]. This is illustrated in Figure 5.

**Figure 6:**
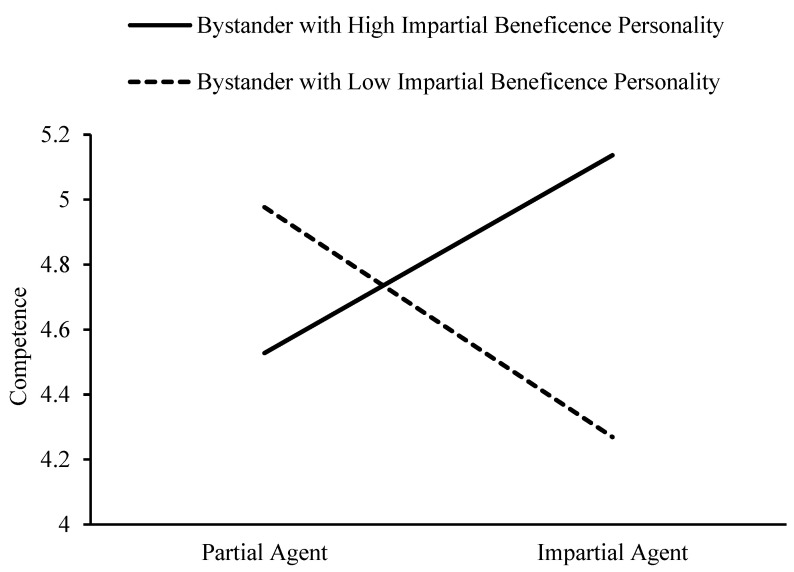
Moderating effect of bystanders’ impartial beneficence personality on the relationship between impartial beneficence and perception of competence.

The impartial beneficence personality of bystander has a significant moderating effect on the influence of impartial beneficence on perception of competence, 95% CI = [0.35, 0.71]. When bystanders exhibit a greater impartial beneficence personality, impartial beneficence positively predict perception of competence, 95% CI = [0.22, 0.73]. When bystanders display a lower impartial beneficence personality, impartial beneficence negatively predict perception of competence, 95% CI = [−0.83, −0.33]. See Figure 6.

## 5. General Discussion

This study explored the mechanisms and boundary conditions through which impartial beneficence influences bystanders’ cooperative behavior across three experiments. The results demonstrated that, compared with partial agent, impartial agent reduced bystanders’ cooperative behavior (Experiment 1). Perception of warmth mediated the influence of impartially beneficence on bystanders’ cooperative behavior (Experiment 2). Additionally, bystanders’ impartially beneficence personality played a moderating role in the mediation model, and this moderation occurred in the pathways from impartial beneficence to bystanders’ cooperative behavior, as well as to perceptions of warmth and competence (Experiment 3).

### 5.1. How Social Perception Acts as a Mediator

This paper verifies the mediating role of perception of warmth in the influence of impartial beneficence on bystanders’ cooperative behavior, whereas perception of competence do not play a mediating role. That is, impartial beneficence reduce bystanders’ cooperative behavior solely by decreasing their perception of warmth. From the perspective of intentions/abilities (Wojciszke & Abele, 2008), people consider only the tendency of impartial beneficence agent to help or harm without considering their ability to help or harm.

According to the Behaviors from Intergroup Affect and Stereotype Map (Cuddy et al., 2007; Guan, 2009), warmth predicts proactive behavior, whereas competence predicts reactive behavior (Wojciszke & Abele, 2008). In impartial beneficence dilemmas, compared with impartial agent, partial agent uphold relational duties, aligning with people’s moral intuitions, therefore, they are perceived as highly warm, prompting bystanders to establish beneficence goals with partial agent. Consequently, bystanders’ cooperative behavior with impartial beneficence is significantly lower than that with partial agent. The influence of impartial beneficence on bystanders’ cooperative behavior supports Relational Regulation Theory of Morality (Rai & Fiske, 2011) and the Morality as Cooperation framework (Curry, 2016). According to the Relational Regulation Theory and the Morality as Cooperation framework, relational duties constitute a universal morality (Curry, 2016; Curry et al., 2019). Even young children believe that they have special moral obligations to their in-group and kin (Rhodes & Chalik, 2013). Impartial beneficence may violate people’s moral intuitions about relational duties, thereby reducing bystanders’ perception of warmth toward impartial beneficence agent and subsequently decreasing their cooperative behavior.

### 5.2. How Impartial Beneficence Personality Influences the Aftereffects of Impartial Beneficence Utilitarian

Study 3 revealed that impartial beneficence influences bystanders’ cooperative behavior through perception of warmth, with bystanders’ impartial beneficence personality serving as a moderating factor. This moderation occurs along the paths from impartial beneficence to cooperative behavior, as well as perceptions of warmth and competence. Briefly, for bystanders with high impartial beneficence personality, impartial beneficence do not significantly predict perception of warmth and cooperative behavior, but they do positively predict perception of competence. Conversely, for bystanders with low impartial beneficence personality, impartial beneficence negatively predict perceptions of warmth, competence, and cooperative behavior. For bystanders with a low impartial beneficence personality, the negative consequences of impartial beneficence align with previous research findings (Everett et al., 2018), indicating a positive correlation between an impartial beneficence personality and the acceptance of impartial beneficence behaviors. For individuals with a low impartial beneficence personality, their acceptance of impartial beneficence judgments is relatively low. According to the PERSOC framework (Back et al., 2011; Rauthmann, 2021), bystanders with a low impartial beneficence personality perceive lower warmth and competence in relation to impartial beneficence, leading to decreased cooperative behavior. In summary, impartial beneficence exhibit stable negative consequences for bystanders with a low impartial beneficence personality.

Additionally, the impartial beneficence personality has opposite moderating effects on the influence of impartial beneficence on perception of competence. Specifically, for bystanders with a low impartial beneficence personality, impartial beneficence negatively predict competence perception, whereas for bystanders with high impartial beneficence personality, impartial beneficence positively predicts competence perception. This may be because impartial beneficence aligns with the impartial thinking of bystanders with high impartial traits, leading to a high acceptance of impartial judgments among them (Bago et al., 2022). In other words, bystanders with high impartial beneficence personality are more likely to approve of impartial agent and thus perceive them as indicative of high competence, whereas bystanders with low impartial beneficence personality are less likely to approve of impartial agent and perceive them as indicative of low competence. The reason why the mediating role of competence in the influence of impartial beneficence on bystanders’ cooperative behavior is not significant is that bystanders’ impartial beneficence personality moderates the impact of impartial beneficence on competence perception. Bystanders with high impartial beneficence personality perceive those making impartial beneficence judgments as having high competence, whereas those with low impartial beneficence perceive those making impartial beneficence judgments as having low competence, resulting in a masking effect.

Accordingly, it can be concluded that the impartial beneficence personality of bystanders plays a significant moderating role in the influence of impartial beneficence on bystanders’ cooperative behavior. The findings of this study can, to some extent, explain the inconsistent results of previous studies on the consequences of impartial beneficence, which overlooked the role of bystanders’ personality traits. According to the PERSOC model, personality and social relationships interact, and personality plays a crucial role in the development of social relationships (Back et al., 2011; Rauthmann, 2021). Therefore, future research on the consequences of utilitarianism in this context should delve deeper into the role of personality variables, such as bystanders’ utilitarian traits.

### 5.3. Theoretical and Practical Implications

#### 5.3.1. Theoretical Implications

This research offers several important theoretical contributions. First, it advances our understanding of the complex interplay between impartial beneficence and social cooperation by identifying specific mechanisms through which impartial beneficence influences bystander behavior. By demonstrating that perceptions of warmth—but not competence—mediate this relationship, our findings extend the dual-dimension model of social cognition (Wojciszke & Abele, 2008) into the domain of impartial beneficence moral judgment and its interpersonal consequences.

Moreover, our research bridges two prominent theoretical frameworks: the two-dimensional model of utilitarian psychology (Kahane et al., 2018) and the interplay of personality and social relationships (PERSOC) framework (Back et al., 2011). The moderating role of impartial beneficence personality provides empirical support for Kahane et al.’s (2018) contention that impartial beneficence represents a distinct dimension of utilitarianism with unique psychological correlates. Additionally, our findings demonstrate how individual differences in moral dispositions shape interpersonal dynamics, consistent with the PERSOC model’s emphasis on personality-relationship reciprocity.

From a cross-cultural perspective, these findings contribute to understanding cultural variations in moral intuitions. While Western philosophical traditions have often championed impartial beneficence as a moral ideal (de Lazari-Radek & Singer, 2017), our results converge with a growing body of cross-cultural research suggesting that relational duties constitute a more universal moral foundation (Curry et al., 2019; Graham et al., 2016). For instance, neglecting kin is judged more harshly than neglecting strangers resonates with anthropological evidence documenting kinship obligations as fundamental moral principles across diverse cultural contexts (Rai & Fiske, 2011). This convergence between our experimental findings and the cross-cultural anthropological data suggests that moral intuitions regarding special obligations to kin may represent an evolutionarily conserved moral psychology that transcends cultural boundaries, rather than merely reflecting Western normative constructions. Supporting this view, Awad et al. (2018) found robust cross-cultural agreement in moral dilemmas, with a widespread preference for protecting close relationships over impartiality. Thus, the observed resistance to impartial beneficence may reflect deeply ingrained moral intuitions that transcend cultural boundaries.

#### 5.3.2. Practical Implications

Our findings have several practical implications. On the one hand, for organizational leaders and decisionmakers, understanding the interpersonal costs of impartial beneficence can inform more effective decision strategies. While impartial approaches may be ethically defensible or even optimal in certain contexts, leaders should anticipate potential reductions in cooperation, particularly among individuals with low impartial beneficence traits.

On the other hand, in conflict resolution and negotiation settings, understanding how different moral orientations affect social perceptions and cooperation could inform more effective mediation strategies. Mediators might adapt their approaches based on the impartial beneficence traits of involved parties to maximize cooperation.

### 5.4. Limitations and Future Directions for Future Research

First, this study examined the consequences of utilitarianism from the perspective of impartial beneficence judgment. Future research should further explore the consequences of utilitarianism from the perspective of impartial beneficence decision making. Second, future studies should delve deeper into the neural mechanisms underlying the influence of impartial beneficence on bystanders’ cooperative behavior. Finally, it is necessary for future research to further explore the connotations and denotations of impartial beneficence and clarify the relationship between impartial beneficence and instrumental harm.

## 6. Conclusions

In conclusion, the current study provides substantial evidence regarding the impact of impartial beneficence on bystander cooperation behavior, offering important insights into the underlying mechanisms and boundary conditions of this relationship. Our findings support three key hypotheses. First, impartial agent reduces bystander cooperation behavior in comparison to partial agent (Hypothesis 1). Second, impartial beneficence affects bystander cooperation behavior via influencing bystanders’ perception of warmth (Hypothesis 2a) than competence. Third, the mediation process is further moderated by the impartial beneficence personality of bystanders, particularly in the pathways from impartial beneficence utilitarian judgment to bystander cooperation behaviors and in shaping warmth and competence perception (Hypothesis 3). These results contribute to the growing literature on utilitarianism and social cooperation. To develop a more comprehensive understanding of the advantages and disadvantages of utilitarian personality traits on utilitarian outcomes, future research should examine more systematically how individual differences in utilitarian orientations influence the interpersonal consequences of utilitarian decision making. Such investigations would further illuminate the complex interplay between moral philosophy, personality traits, and cooperative behavior in social contexts.

## Figures and Tables

**Table 1:** Agents’ judgments and justifications in Experiment 1.

	Partial Agent	Impartial Agent
Zhang Li is a firefighter attempting to rescue individuals from a burning building on the verge of collapse. In the limited time remaining, he can save only one more person. Upon entering a room, Zhang Li finds two trapped individuals whom he immediately recognizes. One is a renowned peace negotiator celebrated for resolving armed conflicts globally; his survival would enable him to continue this vital work, potentially saving many more lives. The other individual is Zhang Li’s mother. Zhang Li faces a critical decision: he must choose whom to save, knowing that the one he does not save will perish.	One of the three partners, Partner A, in the decision-making game believes that Zhang Li should prioritize saving his mother over the famous peace negotiator. A’s rationale is, “I think Zhang Li should save his mother to ensure her survival. Although I recognize that rescuing the peace negotiator has the potential to save more lives, I believe that honoring Zhang Li’s responsibility to his mother holds greater significance.”	One of the three partners, Partner A, in the decision-making game believes that Zhang Li should save the famous peace negotiator rather than his mother. A’s rationale is, “I think Zhang Li should rescue the famous peace negotiator because doing so could potentially save more lives, which I consider to be more important than any duty he owes to his mother.”

**Table 2:** Agents’ judgments and justifications in Experiments 2 and 3.

	Partial Agent	Impartial Agent
Zhang Li is a firefighter attempting to rescue individuals from a burning building on the verge of collapse. In the limited time remaining, he can save only one more person. Upon entering a room, Zhang Li finds two trapped individuals whom he immediately recognizes. One is a renowned peace negotiator celebrated for resolving armed conflicts globally; his survival would enable him to continue this vital work, potentially saving many more lives. The other individual is Zhang Li’s mother. Zhang Li faces a critical decision: he must choose whom to save, knowing that the one he does not save will perish.	Person A, your partner, believes that Zhang Li should prioritize saving his mother over the famous peace negotiator. A’s rationale is, “I think Zhang Li should save his mother to ensure her survival. Although I recognize that rescuing the peace negotiator has the potential to save more lives, I believe that honoring Zhang Li’s responsibility to his mother holds greater significance.”	Person A, your partner, believes that Zhang Li should save the famous peace negotiator rather than his mother. A’s rationale is, “I think Zhang Li should rescue the famous peace negotiator because doing so could potentially save more lives, which I consider to be more important than any duty he owes to his mother.”

**Table 3:** Description and correlation analysis in Study 2.

	*M* ± *SD*	1	2	3	4	5	6	7
Moral Judgment	0.5 ± 0.5							
Warm	4.63 ± 1.70	−0.51 ***						
Competence	4.68 ± 1.24	−0.07	0.32 ***					
Trust Task	5.27 ± 2.97	−0.30 ***	0.46 ***	0.34 ***				
Sex	0.33 ± 0.47	−0.03	−0.07	0.01	0.11 *			
Age	30.73 ± 14.75	−0.04	0.15 **	0.14 **	0.09	0.15 **		
Subjective Social Class	5.35 ± 1.41	0.001	0.19 ***	0.25 ***	0.15 **	0.09	0.17 ***	
Baseline of Trust Task	5.74 ± 2.81	0.03	0.09	0.11 *	0.46 ***	0.05	0.03	0.14 **

Note: * *p* < 0.05, ** *p* < 0.01, *** *p* < 0.001.

**Table 4:** Description and correlation analysis in Study 3.

	*M* ± *SD*	1	2	3	4	5	6	7	8
Impartial Beneficence	0.5 ± 0.5								
Warm	4.72 ± 1.81	−0.43 ***							
Competence	4.74 ± 1.22	−0.02	0.48 ***						
Trust Task	5.92 ± 2.85	−0.23 ***	0.57 ***	0.49 ***					
Sex	0.35 ± 0.48	0.03	0.02	0.04	0.005				
Age	29.75 ± 8.12	0.04	0.09	0.11 *	0.13 **	0.06			
Subjective Social Class	5.56 ± 1.49	0.01	−0.093 *	−0.09	−0.05	−0.07	−0.16 **		
Baseline of Trust Task	6.31 ± 2.58	0.05	−0.001	0.11 *	0.35 ***	0.05	0.14 **	−0.15 **	
Impartial Beneficence Personality	3.47 ± 1.27	0.05	0.15 **	0.10 *	0.16 **	−0.04	0.10 *	0.03	0.10 *

Note: * *p* < 0.05, ** *p* < 0.01, *** *p* < 0.001.

## Data Availability

Data are available upon reasonable request.
